# Prepubertal Female Bartholin's Gland Abscess: A Comprehensive Case Report Study

**DOI:** 10.1155/2024/8812350

**Published:** 2024-04-03

**Authors:** Ahmed Salh Alsayed, Tarek Talaat Harb Elkadi, Mohammed Suliman Alshamari, Azza Tawfik Attallah

**Affiliations:** ^1^Riyadh Armed Forces Hospital, Riyadh Regional Lab, Ulaishah, Riyadh 12746, Saudi Arabia; ^2^King Saud Medical City, Ulaishah, Riyadh 12746, Saudi Arabia

## Abstract

**Introduction:**

Bartholin's gland abscesses are rare in pediatric patients, with limited documented cases. This case report aims to contribute valuable insights into managing this uncommon condition in children.

**Methods:**

A thorough examination and diagnostic workup were conducted on a 4-month-old female infant presenting with labial swelling. Clinical assessment strongly suggested the presence of a Bartholin's gland abscess. A culture of purulent discharge revealed the presence of *Escherichia coli* and Gram-negative *Klebsiella pneumoniae*. Antibiotic susceptibility testing guided the choice of treatment. *Case Presentation*. Despite initial treatment with oral antibiotics and sitz baths, there was limited therapeutic response. Close surveillance under the guidance of a pediatric surgeon continued for two months. Subsequently, surgical excision of the Bartholin gland was performed, and the specimen was sent for pathological examination.

**Results:**

Pathological analysis revealed signs of ulceration and granulation tissue, indicative of a mixed inflammatory response. An eight-month follow-up demonstrated marked improvement and overall well-being in the patient.

**Conclusion:**

This case report underscores the importance of considering Bartholin's gland abscess in diagnosing labial swelling in pediatric patients. The successful outcome achieved through surgical excision and appropriate antibiotic therapy provides valuable insights for potential treatment approaches in similar cases. Continued research and comprehensive studies are essential for establishing optimal treatment protocols for this patient demographic.

## 1. Introduction

Bartholin's gland abscess, a rare occurrence in pediatric patients, has been sparsely documented in the medical literature, with only seven cases reported to date [1]. These abscesses arise from Bartholin's glands, integral to the female reproductive system's physiological lubrication process [2]. Infections, notably by *Escherichia coli*, frequently lead to complications, manifesting as cystic and abscess formations [3]. Treatment options span conservative measures to surgical interventions, underscoring the need for ongoing research to refine therapeutic protocols and enhance patient outcomes [4].

Although unusual in pediatric patients, Bartholin's gland abscesses should be considered in the differential diagnosis for labial swelling in this age group. Conventional management involving incision, drainage, and systemic antibiotics has demonstrated efficacy in pediatric cases [5]. Notably, recurrent instances may necessitate a more extensive intervention, such as the cyst wall excision through marsupialisation [6].

The presented case deviates from the standard approach, as the patient, a four-month-old female infant, underwent a unique course of treatment involving the partial surgical excision of Bartholin's gland cyst wall for resolution. This distinctive approach was implemented after a seven-day course of oral antibiotics, and sitz baths yielded limited therapeutic response, highlighting the complexity of managing Bartholin's gland abscess in pediatric populations. The subsequent clinical decision to pursue surgical removal was made after two months of vigilant monitoring, culminating in the successful excision of the cyst wall [7]. Further study is required to refine treatment protocols and deepen our understanding of Bartholin's gland abscess in pediatric populations, ultimately contributing to improved patient outcomes through evidence-based interventions.

## 2. Case Presentation

### 2.1. Patient Information

A female infant, born at full term and currently 4 months of age, was brought to our attention with a complaint of labial swelling persisting for five days. There was no documented history of urinary tract infection, vulvar discharge, voiding disturbances, or traumatic events. The perinatal period was unremarkable, and there were no reported instances of maternal vaginal discharge or infections during pregnancy.

### 2.2. Clinical Findings

Upon physical examination, the infant displayed signs of distress, though vital signs remained within normal limits. Local examination revealed a tender, soft, fluctuant, and erythematous swelling in the lower half of the left labia minora, measuring approximately 2 cm × 1.5 cm. Notably, the swelling exhibited deep lateral extension, palpable between the labia majora and minora. The clitoris and urethral meatus showed no abnormalities. Clinical assessment strongly suggested the presence of an abscess.

### 2.3. Timeline

This case study outlines a four-month-old female infant who experienced persistent labial swelling. Diagnostic tests identified *Escherichia coli* and *Klebsiella pneumoniae*, leading to antibiotic resistance. Initial treatment included oral antibiotics and sitz baths. After two months, surgical removal of the Bartholin gland was performed. Histological analysis showed granulation tissue formation and ulceration. Eight months of postoperative monitoring showed improvement without recurrence or complications, as shown in Table 1.

### 2.4. Diagnostic Assessment

A sample of the purulent discharge material was collected for culture. The results identified the growth of *Escherichia coli* and Gram-negative *Klebsiella pneumoniae*. Antibiotic susceptibility testing indicated sensitivity to specific antibiotics, including Augmentin, ceftriaxone, cotrimoxazole, and gentamicin.

### 2.5. Therapeutic Intervention

In light of the microbiological findings, the patient was initiated on a seven-day course of oral antibiotics and advised to undergo sitz baths. Despite this treatment approach, there was a limited therapeutic response, prompting the continued close surveillance and follow-up care of the infant under the guidance of a pediatric surgeon.

After two months of diligent monitoring, the pediatric surgeon made the clinical decision to proceed with the surgical removal of the Bartholin gland. The procedure involved excision of the cyst wall. The surgical specimen was meticulously prepared and sent to the anatomical pathology laboratory for further examination.

Upon histological analysis, the specimen exhibited signs of ulceration and the presence of granulation tissue, indicating a mixed inflammatory response characterised by chronic and acute inflammation.

### 2.6. Follow-Up and Outcomes

**Figure 1:**
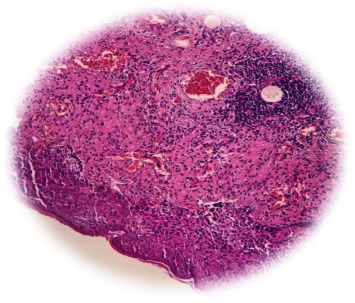
H&E stain shows granulation tissue.

As shown in Figure 1, histopathological examination of the excised Bartholin gland tissue revealed significant granulation tissue formation, indicative of an active tissue repair process characterised by the proliferation of small blood vessels and fibroblasts. The presence of granulation tissue suggests an ongoing inflammatory response.

**Figure 2:**
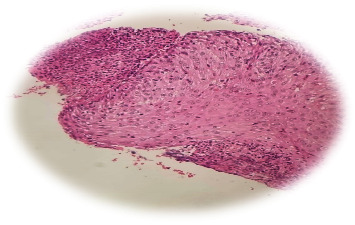
H&E stain shows ulceration tissue.

As shown in Figure 2, the histopathological analysis also revealed areas of ulceration in the excised tissue. Ulceration is characterised by losing epithelial tissue, exposing the underlying layers. This finding suggests a breakdown of the tissue structure, likely due to the inflammatory process and abscess formation.

The patient was closely monitored postoperatively, with regular follow-up visits scheduled. Over eight months, a marked improvement in the patient's condition was observed, demonstrating substantial resolution of the initial complaint. No signs of recurrence or complications were noted during this period.

The surgical intervention, involving the partial excision of the Bartholin gland cyst wall, was instrumental in achieving a favourable outcome for this pediatric patient. This case is valuable to the limited literature on Bartholin's gland abscess in pediatric populations, further emphasising the importance of tailored approaches in managing such cases. The patient's growth and development were monitored during the follow-up period to ensure optimal recovery. Additionally, parental education was provided regarding proper hygiene and signs of potential complications.

Furthermore, it is crucial to note that this case has significant implications for clinical practice, highlighting the importance of considering surgical intervention in cases where conservative measures prove insufficient. By documenting this unique case, we contribute to the collective knowledge base, ultimately enhancing the care provided to pediatric patients with Bartholin's gland abscess.

## 3. Discussion

Bartholin's glands, nestled bilaterally in the posterior vestibule, play a crucial role in maintaining female reproductive health. Their secretory function, akin to Cowper's glands in males, provides essential lubrication for the vaginal vestibule, particularly in response to sexual stimulation [8, 9].

Complications involving Bartholin's glands stem from ductal blockages, leading to the development of cysts [8–10]. These cysts can sometimes become infected, forming Bartholin's gland abscesses, more commonly encountered than ductal cysts [10]. Polymicrobial infections, with *Escherichia coli* as a frequently isolated pathogen, are characteristic of these abscesses. When managing Bartholin's gland abscesses, considering microbiological findings and their corresponding antibiogram is crucial in determining appropriate antibacterial treatment [9, 10].

Bartholin's gland abnormalities can also, though rarely, manifest as carcinomas, representing a small fraction of vulvar cancers; these carcinomas are primarily monitored in postmenopausal women, with adenocarcinoma and squamous cell carcinoma being the most prevalent types. Squamous cell lesions are associated with human papillomavirus, while benign tumours are less frequently observed than carcinoma [9].

Clinical presentation and symptoms of Bartholin's duct cysts may vary. Small cysts can be asymptomatic, while abscesses typically involve inflammation, cellulitis, and lymphangitis [8]. Larger cysts and abscesses lead to significant vulvar pain and swelling, resulting in mobility issues and sexual discomfort, particularly dyspareunia. In some cases, rupture may occur, offering sudden pain relief, and cysts may sometimes mimic inguinal hernias [8, 9].

Distinguishing Bartholin's gland diseases from numerous other labial and vaginal lesions is paramount, which necessitates a meticulous differential diagnosis for abscesses or masses, taking into account various cyst types, hernias, hematomas, cysts of the canal of Nuck, and other conditions such as Gartner's duct cyst and Skene duct urethral cyst. Ensuring an accurate diagnosis guides appropriate treatment [9].

Several methods exist for treating Bartholin's cysts and abscesses; asymptomatic cysts can be left untreated, while incision and drainage offer quick relief but may be prone to recurrence [10]. Sitz baths are recommended for abscesses that rupture spontaneously [8, 10]. Interestingly, in adult females, another conservative option involves using a catheter with an inflatable saline-filled balloon tip, requiring a small incision and leaving the catheter in place for 4–6 weeks, complemented by sitz baths to aid healing. However, it may not be suitable for deep cysts or abscesses; other methods include Foley catheters and Jacobi rings. Marsupialisation is an alternative for cyst drainage but is not ideal for abscesses and can lead to complications like hematoma, dyspareunia, and infection. “Excision by marsupialisation” refers to a surgical technique used to manage Bartholin's gland cysts and abscesses. This procedure involves incision into the cyst or abscess, draining its contents, and then creating a permanent opening (or marsupialisation) to allow continuous drainage and prevent reaccumulation of fluid. While marsupialisation is commonly used for cyst drainage, it may not be suitable for abscesses and can potentially lead to complications such as hematoma, dyspareunia, and infection [8–11]. It is essential to carefully consider the appropriateness of this technique based on the individual characteristics of Bartholin's gland lesion and the patient's clinical presentation.

Comparing the findings of the case report on prepubertal female Bartholin's gland abscess with literature on other rare gynaecological conditions reveals notable similarities and differences. Firstly, the study by Xu et al. [12] on aggressive angiomyxoma in pregnancy mirrors the rarity observed in Bartholin's gland abscess, highlighting diagnostic and management complexities due to their infrequent occurrence. However, while Bartholin's gland abscess predominantly affects pediatric populations, aggressive angiomyxoma typically manifests in reproductive-age women, particularly during pregnancy [12]. Conversely, the study by Gulino et al. [13] on aggressive angiomyxoma of the vulva explores various management strategies, emphasising surgical intervention, differing from the predominantly conservative approach observed in Bartholin's gland abscess cases. Surgical excision was pursued in Bartholin's gland abscess only after failed traditional measures, contrasting with the emphasis on surgical intervention from the outset in aggressive angiomyxoma cases [13].

Additionally, studies by Mendizábal et al. [14] and Gulino et al. [15] on pregnancy-associated melanoma underscore the complexity of managing rare gynaecological conditions during pregnancy. While melanoma poses distinct challenges due to its impact on maternal and perinatal outcomes, it shares commonalities with Bartholin's gland abscess in requiring careful diagnostic and therapeutic strategies tailored to individual patient characteristics [14, 15].

The occurrence of Bartholin's abscess in the pediatric patient cohort, including our case, underscores its rarity in this age group. A comprehensive analysis of the existing literature reveals seven documented cases predominantly caused by *Escherichia coli* [11]. Our case stands out due to the necessity of surgical excision supplementing systemic antibiotics for definitive resolution. In contrast, the remaining cases in this pediatric cohort predominantly underwent incisional drainage coupled with systemic antibiotic therapy. The observed therapeutic diversity emphasises the need for further comprehensive studies to establish optimal treatment protocols and elucidate potential underlying factors contributing to the manifestation of this condition in pediatric patients. This avenue is critical for advancing our understanding of Bartholin's abscess in pediatric populations and enhancing patient outcomes through evidence-based interventions.

Although rare in pediatric patients, Bartholin's gland abscess should be considered in the differential diagnosis for labial swelling in this age group. While the standard approach of simple incision and drainage with broad-spectrum antibiotics typically yields favourable outcomes in pediatric cases, it is worth noting that recurrent instances may require a more extensive intervention, such as excision of the cyst wall through marsupialisation. In our case, the patient underwent a surgical procedure involving excision and removal of the cyst wall for resolution, reinforcing the importance of individualised treatment strategies tailored to each case's unique clinical presentation and characteristics.

Bartholin's gland abscesses in pediatric patients are exceptionally rare, with only a handful of cases documented in the existing literature. Our case adds to this limited body of knowledge and underscores the need for further research on this unique presentation. The successful outcome achieved through surgical excision of the cyst wall highlights the importance of considering individualised treatment approaches. As we accumulate more cases, we can refine our understanding and treatment protocols, ultimately improving outcomes for pediatric patients with Bartholin's gland abscesses.

This case report on a prepubertal female Bartholin's gland abscess highlights the importance of individualised treatment strategies, including surgical intervention when conservative measures fail. It contributes to the limited literature on this rare condition in pediatric patients, emphasising the need for further research to refine treatment protocols. The findings underscore the significance of comprehensive evaluation and tailored approaches for optimal management, offering valuable insights for clinical practice and guiding future research perspectives.

It contributes significant insights by addressing a rare occurrence of Bartholin's gland abscess in a pediatric patient, augmenting the scarce literature on this subject. The successful outcome attained through surgical excision not only underscores the efficacy of this approach but also offers valuable considerations for potential treatment strategies in similar cases in the future, which highlights the importance of continued research and individualised patient care in managing uncommon presentations like pediatric Bartholin's gland abscesses. While the case report provides valuable insights into a rare occurrence of Bartholin's gland abscess in a pediatric patient, it should acknowledge the inherent limitations of a single case report and emphasise the necessity for further research to validate findings and establish optimal treatment protocols.

## 4. Conclusion

In conclusion, this case of a pediatric patient presenting with Bartholin's gland abscess adds to the limited literature on this rare condition in children. The successful outcome achieved through surgical excision of the cyst wall, coupled with appropriate antibiotic therapy, highlights the importance of individualised treatment strategies tailored to the unique clinical presentation and characteristics of each case, which underscores the need for continued scientific inquiry and comprehensive studies to establish optimal treatment protocols for pediatric patients with Bartholin's gland abscesses. As we accumulate more cases, we can refine our understanding and treatment approaches, ultimately improving outcomes for this specific patient demographic.

## Figures and Tables

**Table 1:** Timeline.

Timeline event	Description
Presentation	Full-term female infant, 4 months old, presents with labial swelling persisting for five days
Physical examination	Tender, soft, fluctuant, erythematous swelling measuring approximately 2 cm × 1.5 cm in the lower half of the left labia minora, with deep lateral extension between labia majora and minora
Diagnostic assessment	Culture of purulent discharge: growth of *Escherichia coli* and Gram-negative *Klebsiella pneumoniae*. Antibiotic susceptibility testing indicates sensitivity to specific antibiotics.
Therapeutic intervention	Initiation of a seven-day course of oral antibiotics and sitz baths. Continued close surveillance under pediatric surgeon's guidance due to limited therapeutic response.
Surgical intervention	After two months, surgical removal of the Bartholin gland was performed, involving excision of the cyst wall
Histological analysis	Significant granulation tissue formation and areas of ulceration were observed, indicating a mixed inflammatory response
Follow-up and outcomes	Eight months of postoperative monitoring shows marked improvement with no recurrence or complications, leading to a favourable outcome

## Data Availability

The data used in this study are available upon request.
